# The structure and role of lactone intermediates in linkage-specific sialic acid derivatization reactions

**DOI:** 10.1007/s10719-020-09971-7

**Published:** 2021-01-18

**Authors:** Tamas Pongracz, Aswin Verhoeven, Manfred Wuhrer, Noortje de Haan

**Affiliations:** 1 Center for Proteomics and Metabolomics, Leiden University Medical Center, 2333ZA Leiden, The Netherlands; 2 Present Address: Copenhagen Center for Glycomics, University of Copenhagen, 2200 Copenhagen, Denmark

**Keywords:** Sialic acid, Linkage isomers, Lactone, Mass spectrometry, Nuclear magnetic resonance, Glycomics

## Abstract

Sialic acids occur ubiquitously throughout vertebrate glycomes and often endcap glycans in either α2,3- or α2,6-linkage with diverse biological roles. Linkage-specific sialic acid characterization is increasingly performed by mass spectrometry, aided by differential sialic acid derivatization to discriminate between linkage isomers. Typically, during the first step of such derivatization reactions, in the presence of a carboxyl group activator and a catalyst, α2,3-linked sialic acids condense with the subterminal monosaccharides to form lactones, while α2,6-linked sialic acids form amide or ester derivatives. In a second step, the lactones are converted into amide derivatives. Notably, the structure and role of the lactone intermediates in the reported reactions remained ambiguous, leaving it unclear to which extent the amidation of α2,3-linked sialic acids depended on direct aminolysis of the lactone, rather than lactone hydrolysis and subsequent amidation. In this report, we used mass spectrometry to unravel the role of the lactone intermediate in the amidation of α2,3-linked sialic acids by applying controlled reaction conditions on simple and complex glycan standards. The results unambiguously show that in common sialic acid derivatization protocols prior lactone formation is a prerequisite for the efficient, linkage-specific amidation of α2,3-linked sialic acids, which proceeds predominantly via direct aminolysis. Furthermore, nuclear magnetic resonance spectroscopy confirmed that exclusively the C2 lactone intermediate is formed on a sialyllactose standard. These insights allow a more rationalized method development for linkage-specific sialic derivatization in the future.

**Supplementary Information:**

The online version contains supplementary material available at 10.1007/s10719-020-09971-7.

## Introduction

Protein glycosylation is a ubiquitous co- and post-translational modification, which has lately received considerable attention given its relevance in a multitude of biological processes [1, 2]. Glycosylation affects folding and solubility of glycoproteins, and changes in response to diverse environmental cues [3, 4]. Sialic acids are monosaccharides which are end-capping glycans, where they play important roles in either masking the glycoprotein from its surroundings or by mediating interaction with glycan-binding proteins [5]. The importance of sialylated glycans is exemplified by their broad involvement in host-pathogen interactions [6–8], glycoprotein half-life in the circulation [9], lymphocyte homing [10], inflammation [11] and tumor development [12, 13]. Of note, sialic acids typically terminate glycans in α2,3- or α2,6-linkages, adding an additional layer of functional complexity to the glycan moiety [5].

**Fig. 1:**
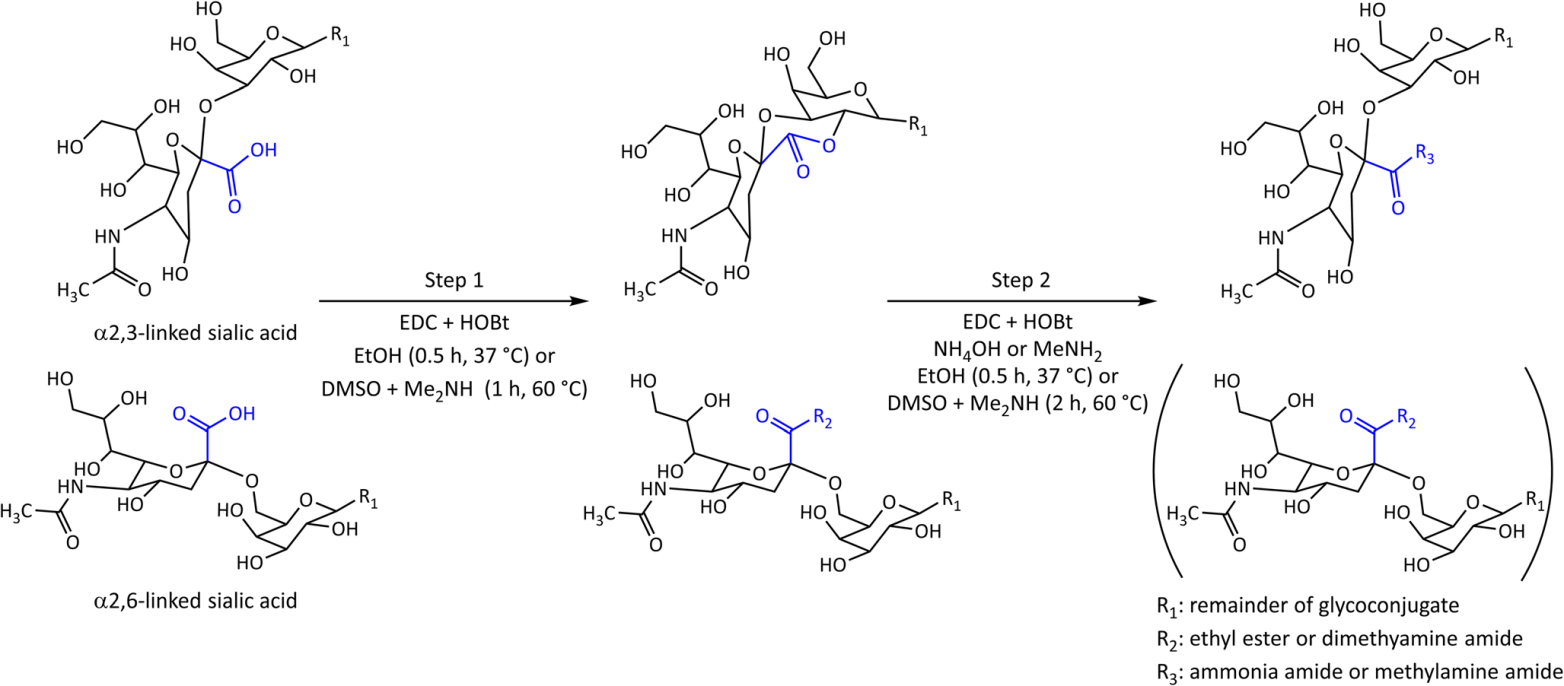
Schematic representation of common one-pot in-solution linkage-specific derivatization approaches for sialylated glycoconjugates. In the first step, α2,3-linked sialic acids form a lactone with the subterminal galactose, while α2,6-linked sialic acids are subjected to ethyl esterification or Me_2_NH (dimethylamine) amidation. In the second step, the lactone undergoes mainly direct aminolysis by NH_3_ (ammonia) or MeNH_2_ (methylamine), and the α2,6-sialyllactose derivative remains unchanged

To assess sialic acid linkages qualitatively and quantitatively, an array of techniques has been developed relying on the differential derivatization of the carboxyl groups of the isomers and their detection by mass spectrometry (MS) [6, 14–20]. In the presence of a carboxylic acid activator (such as 1-ethyl-3-(3-dimethylaminopropyl) carbodiimide; EDC [21]) and a catalyst (such as 1-hydroxybenzotriazole; HOBt [22]) α2,6-linked sialic acids react with added amines or alcohols to form amide or ester derivatives, respectively. In contrast, α2,3-linked sialic acids form lactones under the same conditions **(**Fig. 1**)** [14, 20]. Because of the limited stability of the lactones, a second reaction step is often included to convert them into amide products [15, 23].

Four different approaches have been described to get from lactonized α2,3-linked sialic acids to a stable end product: 1) Lactones were purified and hydrolyzed under alkaline conditions before treating them with a second nucleophile in the presence of a carboxyl activator [17, 19]. 2) Lactones were subjected to a mild clean-up step and amidated in a ring-opening reaction in the sole presence of an amine (aminolysis) [24]. 3) The second nucleophile was directly added to the reaction mixture to obtain the stable derivative of the α2,3-linked sialic acids [16, 23, 25]. 4) Lactones were purified under mild conditions at neutral pH and treated with a second nucleophile in the presence of a carboxyl activator and catalyst [16]. While the first approach relies on the reaction of the unmodified carboxyl group and the second relies on direct aminolysis of the lactone, for approaches 3 and 4 the reaction path remains unclear. Specifically, one may speculate that direct lactone aminolysis may results in the stable derivative of α2,3-linked sialic acids in these cases. Alternatively, lactones may be opened by hydrolysis followed by derivatization of the free carboxyl group [26]. Research in the field of linkage-specific sialic acid derivatization has been abundant, with the methodological advances realized as of today summarized in two succinct reviews [26, 27]. However, while one-pot reactions such as approach 3 (Fig. 1) are gaining popularity due to their ease of use and versatility, the conversion of the lactone intermediate into a stable derivative – albeit a key part of the approach – is insufficiently understood, thereby hampering the further design and optimization of the approach.

In order to address this gap in understanding, we here studied the lactone dependency of the amidation step of α2,3-sialylated glycans in one-pot sialic acid derivatization approaches. For this, we used an α2,3-sialyllactitol standard and α2,3-sialylated, complex-type *N*-glycans of recombinant human erythropoietin (rhEPO). In addition, we employed NMR spectroscopy to characterize the configuration of the lactone for an α2,3-sialyllactose standard under representative conditions used in differential sialic acid derivatization.

## Materials and methods

### Chemicals, reagents and enzymes

All materials and reagents used in this study were of analytical grade and purchased from commercial suppliers. Type I Ultrapure Water was produced by an ELGA Purelab Ultra system (Elga LabWater, High Wycombe, UK) and used throughout. Ethanol (EtOH), sodium hydroxide (NaOH), SDS (sodium dodecyl sulfate), TFA (trifluoroacetic acid) and disodium hydrogen phosphate dihydrate Na_2_HPO_4_∙2H_2_O), potassium dihydrogen phosphate (KH_2_PO_4_), sodium borohydride (NaBH_4_), sodium chloride (NaCl) and Dowex 50 W X8 cation exchange resin were purchased from Merck (Darmstadt, Germany). Glacial acetic acid and potassium hydroxide were obtained from Honeywell Fluka (Charlotte, NC). 1-Hydroxybenzotriazole (HOBt) hydrate, ortho-phosphoric acid, 40 wt.% aqueous methylamine (MeNH_2_), 40 wt.% aqueous dimethylamine (Me_2_NH), 28–30 wt.% aqueous ammonium hydroxide (NH_4_OH), anhydrous DMSO and a mixture of 2,5-dihydroxybenzoic acid and 2-hydroxy-5-methoxybenzoic acid (super-DHB; sDHB) were obtained from Sigma-Aldrich (Steinheim, Germany), while 1-ethyl-3-(3-dimethylaminopropyl) carbodiimide (EDC) hydrochloride was acquired from Fluorochem (Hadfield, UK). HPLC Supra-gradient acetonitrile (ACN) originated from Biosolve BV (Valkenswaard, Netherlands), and Peptide Calibration Mix II from Bruker Daltonics (Bremen, Germany). Recombinant peptide-N-glycosidase F (PNGase F) was purchased from Roche Diagnostics (Mannheim, Germany). Acidic PBS (pH 5.6) was prepared as described previously [24]. The *N*-glycan release mixture was composed of 10 μL 4% Nonidet P-40 supplement (VWR International, Solon, OH), 10 μL 5x acidic PBS, and 1 U PNGase F. 2,2,3,3-D4 sodium trimethylsilylproprionate (TSP) was obtained from Cambridge Isotope Laboratories (Tewksburry, MA), and deuterated water (D_2_O) (99.8 atom % D) from Cortecnet (Voisins-le-Bretonneux, France).

### Samples

Commercially available sialyllactose (SL) and sialylLacNAc (SLN) standards with known sialic acid linkage (α2,3 or α2,6) were obtained from Carbosynth (Compton, UK). SL and SLN standards were dissolved to a final concentration of 10 μg/μL. 2,3-SL samples for NMR analysis were prepared at a final concentration of 5 mg/μL (reference NMR sample without sialic acid derivatization) or 1 mg/μL (with sialic acid derivatization) in D_2_O. The NMR sample with sialic acid derivatization was prepared in triplicate, pooled and added up to 200 μL final volume with D_2_O before analysis. Chinese hamster ovary (CHO) cell culture-derived rhEPO was kindly provided by Roche Diagnostics (Penzberg, Germany), and immunoglobulin G (IgG) affinity purified from normal human plasma was obtained from Athens Research & Technology Inc. (Athens, GA).

### Reduction of sialyllactose

Sodium borohydride-reduced SL (sialyllactitol) standards were prepared according to established procedures [28], but by replacing methanol with isopropanol throughout to prevent methyl esterification of the carboxylic acids. The dried samples were reconstituted in water to a final concentration of 10 μg/μL.

### PNGase F *N*-glycan release

*N*-glycans from rhEPO and IgG were released in acidic PBS [21]. Briefly, 20 μL 2% SDS was added to 10 μL rhEPO (50 μg) or IgG (41.8 μg) standard and shaken for 5 min on a horizontal shaking platform at 1350 rpm (rotary motion of 1.5 mm) followed by 10 min incubation at 60 °C. The sample was allowed to come to room temperature before the addition of 20 μL release mixture. The samples were shaken for 5 min at 1350 rpm, followed by overnight incubation at 37 °C, and stored at −20 °C until sialic acid derivatization.

### Preparation of sialic acid derivatization reagents

The ethyl esterification reagent (EE reagent) was prepared by dissolving EDC and HOBt in EtOH to a final concentration of 0.25 M of both chemicals. The dimethylamine amidation reagent (DMA reagent) was prepared by dissolving EDC and HOBt and adding 40% Me_2_NH to DMSO in a final concentration of 0.25 and 0.5 and 0.25 M, respectively. The control-reagents were 100% EtOH and 0.25 M Me_2_NH in DMSO. The pH of all reagents was measured in triplicates using narrow range pH indicator strips, after ten times dilution in water (Supplementary Table 1).

### Linkage-specific sialic acid derivatization

Ethyl esterification (EE) and Me_2_NH amidation (DMA) were performed according to established procedures [14, 20]. Briefly, 20 μL EE reagent or DMA reagent was added to the wells of a 96-well NUNC V-bottom plate (Thermo Scientific, Waltham, MA). Then, the reactions were performed by adding 1 μL of one of the SL standards (10 μg), sialyllactitol (10 μg) or released *N*-glycan mixture, followed by 30 or 60 min incubation at 37 or 60 °C for EE and DMA, respectively. In case of ethyl esterification + NH_3_ amidation (EE + AA) [25, 29], ethyl esterification + MeNH_2_ amidation (EE + MA), Me_2_NH + NH_3_ amidation (DMA + AA) [23], or Me_2_NH amidation + MeNH_2_ amidation (DMA + MA), 4 μL of NH_4_OH (final concentration of 1.34 M) or MeNH_2_ (final concentration of 1.83 M) were added to the reaction mixture followed by further incubation for 30 min (in case of EE + AA and EE + MA) or 120 min (in case of DMA + AA and DMA + MA). The direct amidation reagents were prepared by adding 4 μL of NH_4_OH or MeNH_2_ to the reagents right before sample addition. All samples were prepared in triplicate.

### Cotton HILIC SPE

Cotton HILIC SPE of sialic acid-derivatized SL and released *N*-glycans was performed according to established procedures described elsewhere [22, 30]. Briefly, following derivatization, ACN was added to the reaction mixture to 50% *v*/v (EE ± AA or MA) or 85% v/v (DMA ± AA or MA) followed by cotton-HILIC purification. The retained glycans were eluted in 10 μL water. For NMR analysis, the sample was washed with and eluted into D_2_O. To prepare the glycan samples for MALDI-TOF-MS, 1 μL sDHB (5 mg/mL in 50% ACN with 1 mM NaOH), while for MALDI-FT-ICR-MS, 1 μL sDHB (5 mg/mL in 50% ACN with 0.1 mM NaOH) was spotted on an AnchorChip 800/384 TF MALDI target (Bruker Daltonics) topped with 1 μL HILIC enriched glycans. The spots were left to dry by air.

### MALDI-TOF-MS

MALDI-TOF-MS analyses were performed on an UltrafleXtreme mass spectrometer equipped with a Smartbeam-II laser (Bruker Daltonics, Bremen, Germany). Spectra were acquired in reflectron positive mode collecting a total of 10,000 laser shots at a laser frequency of 1000 Hz, using 25 kV acceleration voltage. Prior to measurement, the instrument was calibrated with Peptide Calibration Mix II (Bruker Daltonics). An *m/z* range of 300–1000 was used for all SL, SLN, and sialyllactitol measurements.

### MALDI-FT-ICR-MS

MALDI-FT-ICR-MS analyses were performed on a 15 T solariX XR FT-ICR mass spectrometer equipped with a CombiSource, a ParaCell and a Smartbeam-II laser (Bruker Daltonics, Bremen, Germany). Spectra were acquired in positive mode. Prior to measurement, the instrument was calibrated using CsI cluster masses for the analysis of methyl esterified α2,3-sialyllactitol, or Peptide Calibration Mix II (Bruker Daltonics) for the measurement of rhEPO and IgG released *N*-glycans. For each spot, an average spectrum was obtained from the acquisition of 10 spectra in the *m/z* range of 153–1000 for sialyllactitol or 1011–5000 for released *N*-glycans, using 1 M data points.

### MS data analysis

Data analysis was performed with the in-house developed software MassyTools (version number 1.02200129a) [31]. Internal calibration was performed based on a selected calibrant list (Supplementary Table 2–4), followed by targeted data extraction using predefined glycan compositions. Data quality control was performed based on quality control parameters (isotopic pattern quality (IPQ), ppm error, and S/N). Total area normalization of the extracted glycan signals passing quality control criteria (IPQ < 0.2; ppm deviation < 15 ppm; S/*N* > 3) was performed for each spectrum (Supplementary Table 5–7). Analytes resulting in overlapping signals (e.g. the NH_3_ amidated SL or sialyllactitol and their unmodified counterpart) were corrected based on the theoretical overlap between their isotopic patterns. Averages and SDs were calculated from triplicate measurements using Microsoft Excel.

### NMR spectroscopy

All NMR data was recorded on a Bruker AVANCE II spectrometer equipped with a 14.1 T magnet and a 5 mm TCI cryogenic probe head and a z-gradient system. The samples were manually injected into disposable 5 mm SampleJet NMR tubes and sealed with a closed cap. A Bruker SampleJet system was used for sample insertion, removal and temporary storage. In the SampleJet the samples were kept at a temperature of 6 °C. The temperature for the NMR measurements was 300 K, which was carefully calibrated using a fresh methanol-d4 sample. TSP was used as chemical shift reference. All sample were measured in an 1D NOESY ^1^H NMR experiment with 25 Hz water presaturation and a relaxation delay of 4 s. The reference α2,3-sialyllactose was measured by accumulating 4 scans, for the lactone sample 256 scans were collected. The lactonized α2,3-sialyllactose was analyzed directly after preparation, as well as after 16 h of storage at 300 K. The raw NMR data was processed and analyzed in Bruker TopSpin 3.0.

## Results and discussion

Here, we present a set of experiments performed to unravel the role of the lactone intermediate in the amidation during linkage-specific sialic acid derivatization. We further present the structural characterization of the lactone intermediate formed with common linkage-specific sialic acid derivatization conditions.

### The role of the lactone intermediate in the amidation of sialyllactitol

The lactone dependency of the amidation reaction was investigated using an α2,3-linked sialyllactitol standard. Sialyllactitol was chosen as a suitable standard as it lacked the reducing end aldehyde, thereby avoiding reducing end associated side reactions (Supplementary Fig. 1) (Supplementary Table 5). In order to avoid methyl ester side products arising during sialyllactitol preparation, the methanol used in the original protocol during cleanup [28] was replaced by isopropanol (Supplementary Fig. 2 and 3).

**Fig. 2:**
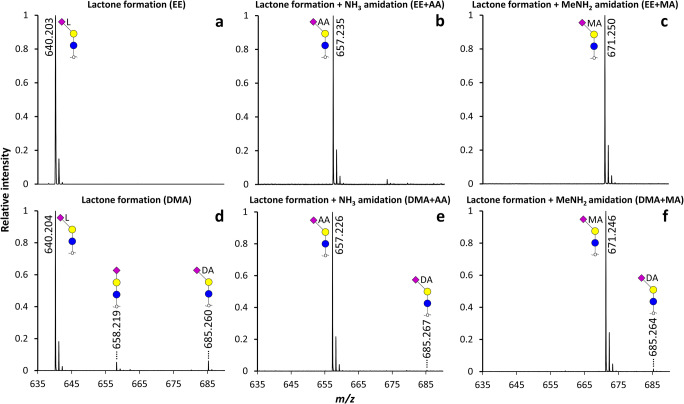
Representative MALDI-TOF-MS spectra showing the modifications induced on an α2,3-sialyllactitol standard under native reaction conditions in EtOH **(a-c)** or DMSO **(d-f)**. Symbols indicate the monosaccharide residues glucose (blue circle), galactose (yellow circle) and *N*-acetylneuraminic acid (purple diamond). In case of derivatized sialic acids, an α2,3-linkage is depicted with a left angle, with lactonization indicated by L, NH_3_ amidation by AA, MeNH_2_ amidation by MA, and Me_2_NH amidation by DA next to the sialic acid residue. Non-derivatized sialic acids are depicted without an angle. All species were detected as [M + Na]^+^

**Fig. 3:**
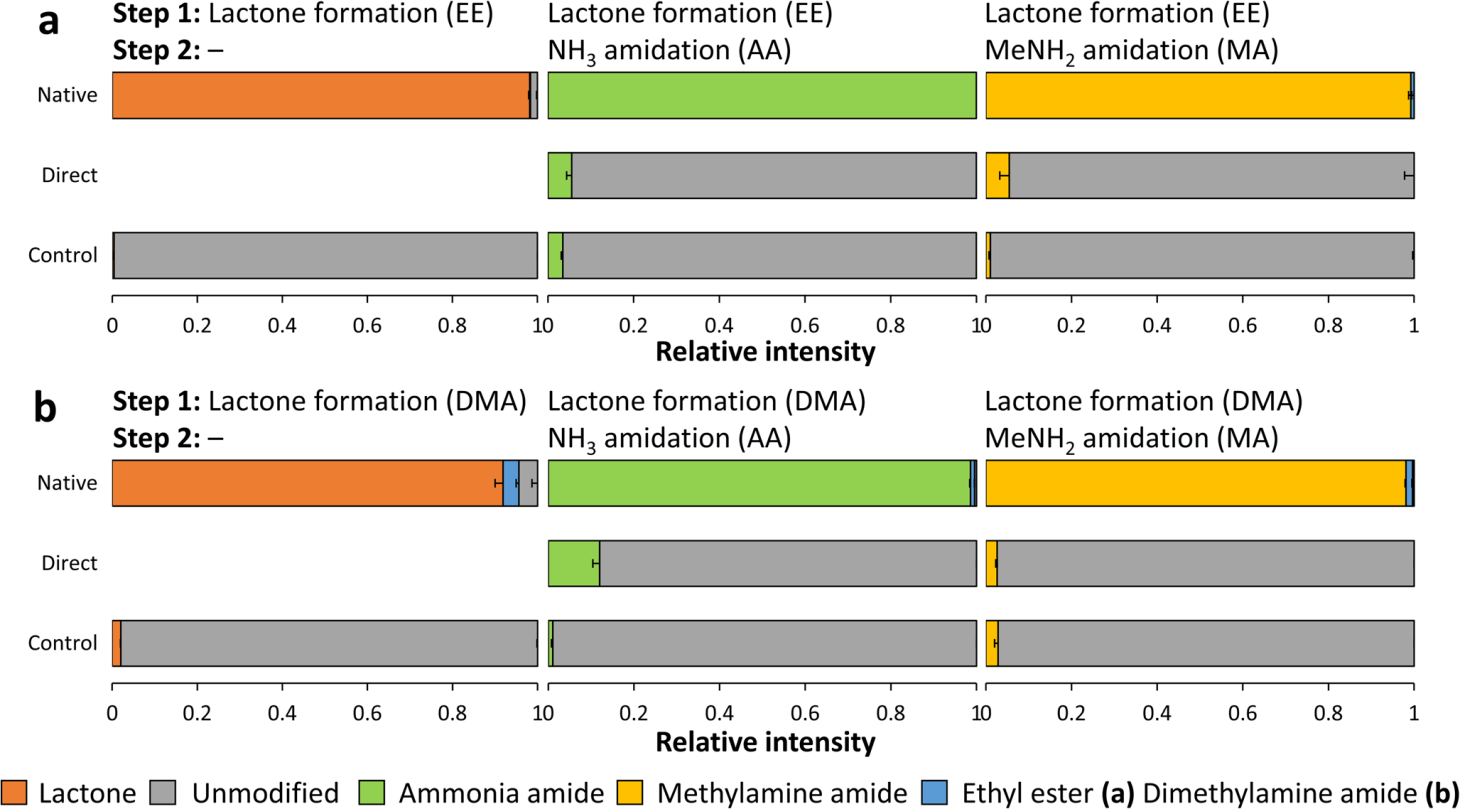
Bar graphs illustrating the role of the lactone intermediate on the NH_3_ amidation and MeNH_2_ amidation step in EtOH **(a)** or DMSO **(b)** using an α2,3-sialyllactitol standard. Native reaction conditions refer to the application of either EE or DMA reagent in the first step of the reaction and the addition of NH_4_OH or MeNH_2_ in the second step. The direct conditions mimic the NH_3_ amidation or MeNH_2_ amidation step by the immediate addition of NH_4_OH or MeNH_2_ in the first step. The control conditions refer to the use of control-reagents without EDC and HOBt following the two-step reaction. The average and SDs for triplicate measurements are shown as stacked bars and error bars, respectively. The pH of the conditions was evaluated in triplicate using narrow range pH indicator strips (Supplementary Table 1)

To resolve the ambiguity around the lactone dependency of amidation, first the lactone formation of α2,3-sialyllactitol was promoted based on preceding reports using the EE or DMA reagent [22, 23]. Using both conditions, α2,3-sialyllactitol underwent near complete intramolecular water loss (*m/z* 640.204) (Fig. 2a, d and Fig. 3) (Supplementary Table 6). In the second step, conditions were set as to promote NH_3_ amidation [23, 25] or MeNH_2_ amidation [16, 24]. High conversion efficiency was observed for the lactonized species following all conditions resulting in the ammonia amide at *m/z* 657.235, and methylamide at *m/z* 671.250 (Fig. 2 and Fig. 3**)** (Supplementary Table 6). While non-selective conversion of the standard was scarcely observed using EE reagent (0.2 ± 0.02% ethyl ester formation for α2,3-sialyllactitol; *m/z* 686.243), in case of DMA reagent, the Me_2_NH amidated α2,3-sialyllactitol by-product showed 3.7 ± 0.6% relative abundance (*m/z* 685.260 (Fig. 2d and Fig. 3)). This can be explained by the higher nucleophilicity of Me_2_NH as compared to EtOH, leading to higher rates of misconversion, yet the linkage-specific derivatization of minor amounts of a potential α2,6-sialyllactitol contaminant may likewise contribute to this signal [22]. The relative abundance of the Me_2_NH amidated by-product decreased to 0.9 ± 0.1% and 1.5% ± 0.1% after NH_3_ amidation or MeNH_2_ amidation, respectively. The minor relative abundance deviations between the lactone, ammonia amide and methylamide forms may be caused by differences in ionization efficiency of the reaction products [32] and/or from subtle response factor differences of the MALDI-TOF-MS detector [33].

Next, the alkaline NH_3_ or MeNH_2_ amidation conditions used in the second step of common protocols [16, 23, 25] described above were directly applied on the untreated α2,3-linked standard (Supplementary Table 6). This resulted in only a small proportion of the α2,3-sialyllactitol to be amidated using either EE or DMA reagent in combination with NH_4_OH or MeNH_2_. The highest conversion was observed for DMA + AA, resulting in 12.0 ± 1.6% NH_3_ amidated product (Fig. 3 “Direct”) (Supplementary Fig. 4 and 5) (Supplementary Table 6). This indicates that prior lactone formation is essential to complete amidation of α2,3-linked sialic acids under the described conditions using EDC and HOBt (or analogues thereof). The alkaline reaction conditions did also not result in any modification of the α2,6-sialyllactitol standards run in parallel, which is in accordance with the acidic pH optimum of EDC reactivity [6, 34] (Supplementary Fig. 6 and 7).

To further confirm the lactone dependence of high-pH α2,3-linked sialic acid amidation, reactions identical to the native ones were performed, but excluding the presence of EDC and HOBt. These conditions were not supposed to induce lactone formation and indeed resulted in only trace amount of the lactonized α2,3-sialyllactitol standard (Fig. 3 “Control”) (Supplementary Fig. 4 and 5) (Supplementary Table 6), which in addition rather originated from the reducing step (Supplementary Fig. 2). Correspondingly low amidation rates were detected, independently from the chosen solvent and nucleophile (Fig. 3) (Supplementary Fig. 4 and 5) (Supplementary Table 6). In addition, no modifications were observed on the α2,6-linked sialyllactitol controls (Supplementary Fig. 6 and 7).

From these results one can conclude that under common one-pot linkage-specific sialic acid derivatization conditions, prior lactone formation is a prerequisite for complete, linkage-specific amidation of α2,3-linked sialyllactitol. The reaction proceeds mainly via direct aminolysis and independently from EDC and HOBt. At the same time, free carboxylic acids of α2,3-sialyllactitol react to a minor extent with NH_3_ and MeNH_2,_ exclusively in the presence of EDC and HOBt. Thus, both proposed mechanisms potentially co-occur, with direct aminolysis being essential for complete amidation of the α2,3-linked sialic acids (Fig. 3**)**. In view of these results, the conventional incubation time in the second step of such reactions [16, 17, 19, 23, 25] may significantly be shortened, as direct aminolysis has been described as an instantaneous reaction [24]. Furthermore, for protocols where the lactonized species are purified under mild conditions at neutral pH prior to treatment with a second nucleophile [16], the re-addition of carboxylic acid activators is not required. While, the direct aminolysis was here demonstrated for α2,3 linked sialic acids only, the proposed mechanisms are also likely to be true for α2,8- and α2,9-linked sialic acids, which are as well prone to form lactones [35]. Lactone mediated amidation was shown for α2,8-linked sialic acids on glycosphingolipid glycans previously [24].

For further investigations, exclusively the DMA + MA conditions were used. As opposed to the ethyl esterified α2,6-sialyllactitol the Me_2_NH amidated product was not affected by alkaline cleavage using NH_4_OH or MeNH_2_ (Supplementary Fig. 6–8) (Supplementary Table 6). Furthermore, the use of MeNH_2_ and not NH_4_OH for the second reaction step resulted in easier interpretable spectra by preventing the overlap between unmodified and NH_3_ amidated species (Supplementary Fig. 4).

### The role of the lactone intermediate in the amidation of complex *N*-glycans

**Fig. 4:**
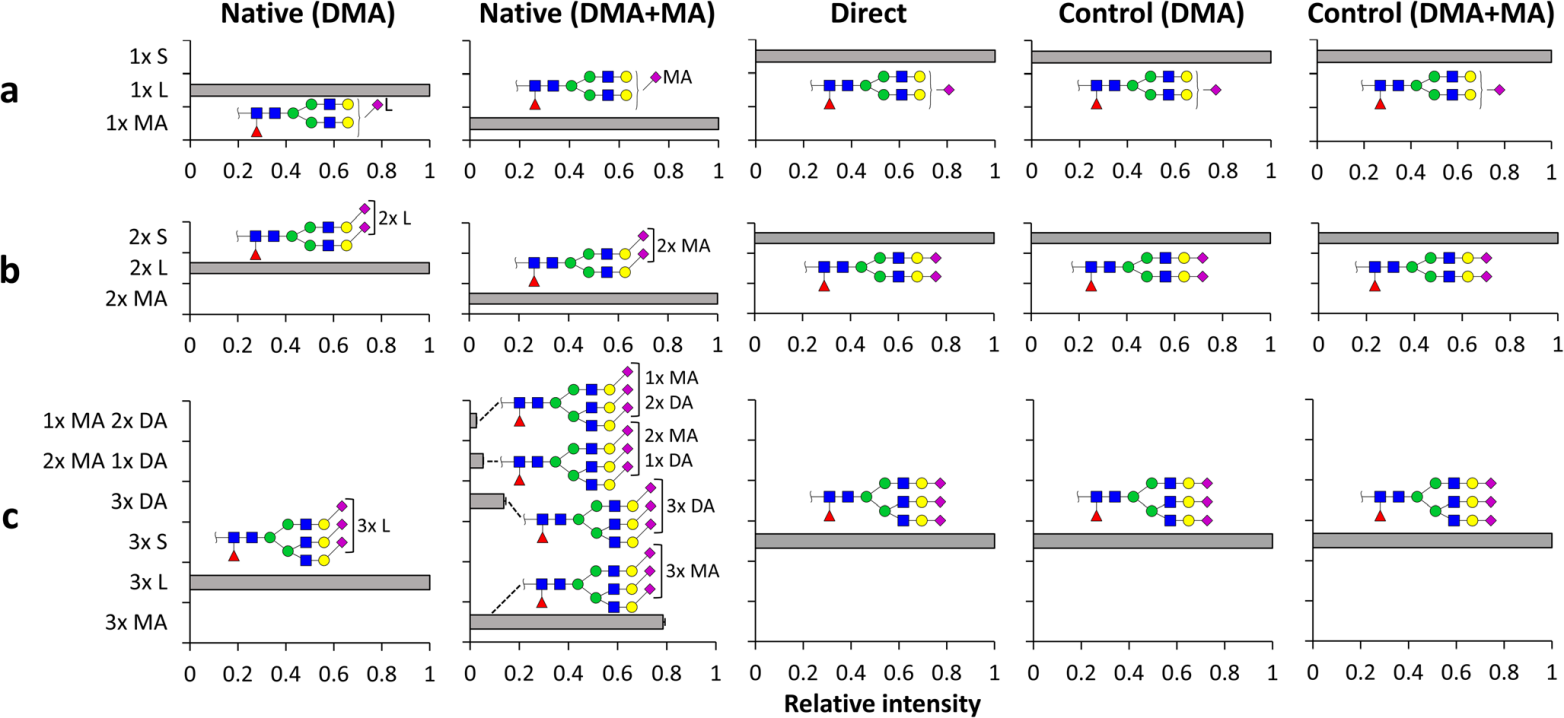
Reaction products observed for α2,3-linked *N*-glycans from rhEPO under different conditions. **(a)** H5N4F1S1; **(b)** H5N4F1S2 and **(c)** H6N5F1S3 are the gross glycan compositions evaluated. Abbreviations indicate H (hexose); N (*N*-acetylgalactosamine); F (fucose); S (*N*-acetylneuraminic acid). Symbols indicate the monosaccharide residues mannose (green circle), galactose (yellow circle), *N*-acetylgalactosamine (blue square), fucose (red triangle), and *N*-acetylneuraminic acid (purple diamond). In case of derivatized sialic acids, an α2,3-linkage is depicted with a left angle, with lactonization indicated by L, MeNH_2_ amidation by MA, and Me_2_NH amidation by DA next to the sialic acid residue. Non-derivatized α2,3-sialic acids are depicted without an angle. The average and SDs for triplicate measurements are shown as bars and error bars, respectively (Supplementary Table 7)

To validate if our findings hold true for more complex sialylated glycans, α2,3-sialylated *N*-glycans released from rhEPO were subjected to linkage-specific sialic acid derivatization using the DMA + MA approach. The most abundant glycans were singly fucosylated, highly sialylated, partially *O*-acetylated complex-type *N*-glycans with a varying number of LacNAc units, as described previously [33] (Supplementary Fig. 9). In the first step of the DMA + MA reaction, lactonization was observed for the sialic acids on the mono-, di-, and trisialylated species H5N4F1S1, H5N4F1S2 and H6N5F1S3 (Fig. 4) (Supplementary Table 7). In the second step of native reaction conditions, fully MeNH_2_ amidated analytes were observed for mono- and disialylated structures with only unquantifiable traces (S/*N* < 3) of misconversion due to Me_2_NH amidation (Fig. 4a, b) (Supplementary Table 7). On the other hand, 78.5 ± 0.8% of the trisialylated analytes underwent MeNH_2_ amidation (Fig. 4c) (Supplementary Table 7). Of note, this substrate was in addition found in singly, doubly, as well triply Me_2_NH amidated form, induced by the remaining Me_2_NH from the first step (Fig. 4c) (Supplementary Table 7), as previously reported [16].

When subjected to direct high pH MeNH_2_ amidation conditions, none of the analytes were MeNH_2_ amidated. Similarly as found for the sialyllactitol standards, this indicates that prior lactone formation is essential for the amidation of α2,3-linked sialic acids under the reported conditions. Likewise, with the application of control-reagents deprived from EDC and HOBt, all analytes remained unmodified, indicating that no carboxyl group modification takes place without prior carboxyl activation (Fig. 4). Of note, the instability of unmodified sialic acids lead to in-source desialylation during MALDI-MS and *N*-glycan acetylation is partly lost under the described reaction conditions, hence causing an underestimation of these species (Supplementary Fig. 9).

To ensure the sialic acid linkage-specificity of the reaction conditions, the described experimental conditions were in addition performed using released glycans deriving from an IgG standard (Supplementary Fig. 10), for which sialylated species have been reported to be almost exclusively α2,6-linked [36]. Under DMA + MA conditions, the detected mono- and disialylated species were entirely Me_2_NH amidated (Supplementary Fig. 11) (Supplementary Table 7). The obtained glycosylation profiles and relative sialic acid abundancies for IgG as well as rhEPO were highly similar to what was previously reported for these glycoproteins [33, 36].

### Structural characterization of the lactone

**Fig. 5:**
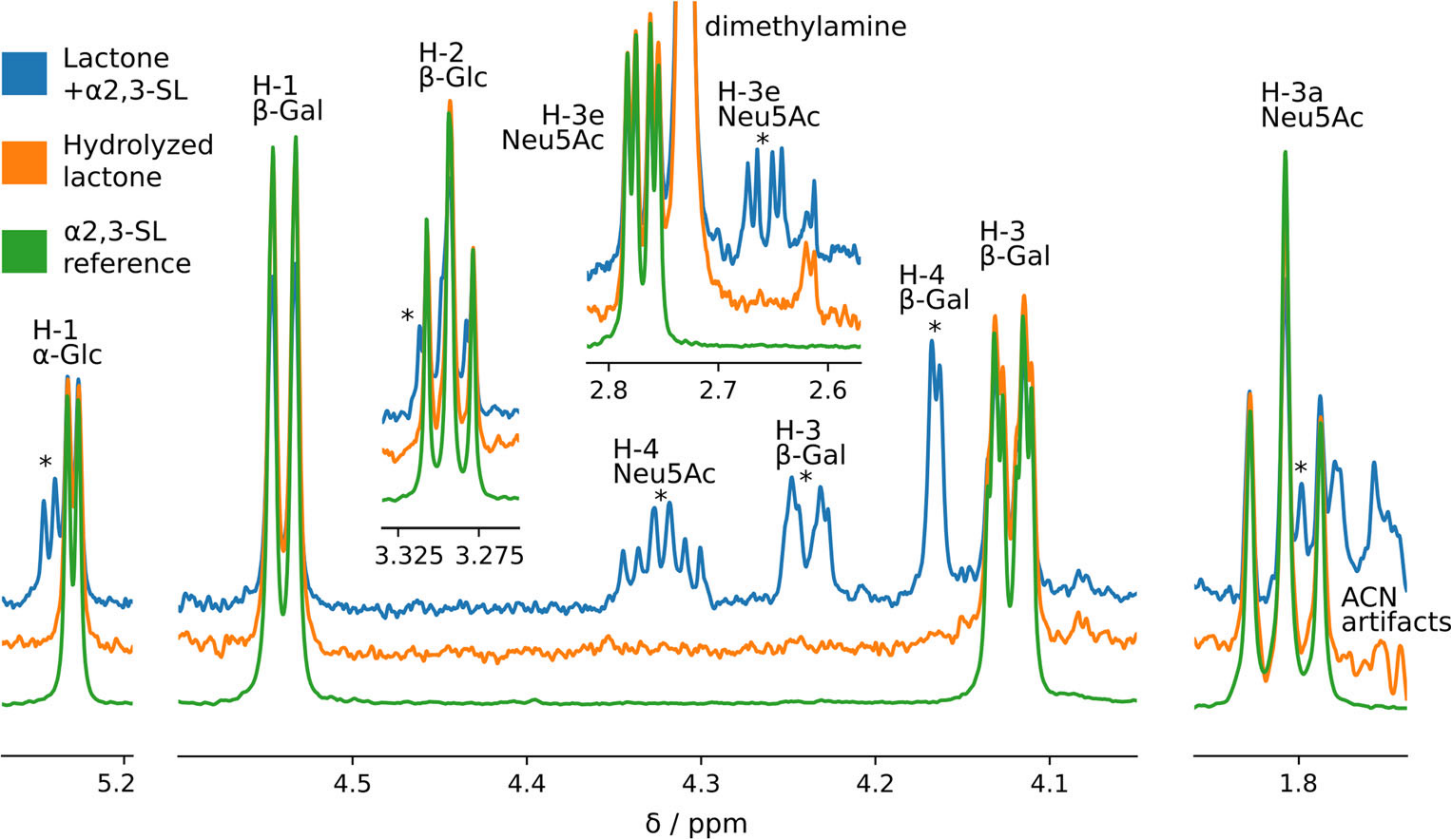
1D ^1^H NMR spectra of the reference α2,3-sialyllactose standard and its C2 lactone derivative. Overlaid spectra of the reference α2,3-sialyllactose standard (green), the same standard after subjected to lactone formation (blue), and the lactonized standard 16 h after the first measurement (orange). The lactone peaks are marked with an asterisk. Contaminant peaks of dimethylamine and ACN originate from cotton HILIC SPE washing steps

While NMR spectroscopy has previously been utilized to characterize sialyllactones of varying origin, the reaction products obtained with carboxylic acid activators commonly used in sialic acid derivatization, especially in the presence of a catalyst and a nucleophile, have not yet been elucidated in a similar manner [37, 38]. To address this, we first recorded a 1D ^1^H NMR spectrum of α2,3-SL dissolved in D_2_O as a reference spectrum (Fig. 5). Second, lactonization of α2,3-SL was performed under DMA conditions as described above, and the products were analyzed by 1D ^1^H NMR. Not all peaks of α2,3-SL were properly resolved in the 1D NMR spectrum, due to the strong coupling effects and resulting overlap in the region between 3.55 and 4.01 ppm. However, the peaks that were resolved allowed the identification of the lactone and to follow the hydrolysis product over time (Fig. 5). Exclusively the C2 lactone was detected, indicated by the diagnostic ppms (Supplementary Table 8). As determined from the peak integrals, a significant amount (64%) of unmodified α2,3-SL was detected already at the first measurement timepoint. This was likely a result of sample hydrolysis, as a measurement time of approximately 1 h was required to obtain sufficient sensitivity. When remeasuring the sample after 16 h of storage at 300 K, only unmodified α2,3-SL was detectable, indicating completed lactone hydrolysis. Of note, the C4 lactone was not detected, either due to its low abundance or rather quick hydrolysis during analysis. However, rapid hydrolysis of the C4 lactone is unlikely, as it has been reported to be more resistant to hydrolysis in an α2,3-SL structure, as opposed to the C2 lactone [39]. The results are in alignment with previous reports on glacial acetic acid induced lactonization of α2,3-SL, showing that this structure mainly results in a C2 lactone [39]. Likewise, ganglioside GM3 lactonization was found to result in a C2 lactone [40]. In contrast, lactonization of a synthetic sialyl T benzyl glycoside resulted in C2 and C4 lactones in 3:2 ratio and a synthetic MUC1 glycopeptide with a sialyl T glycan moiety mainly yielded C4 lactones following glacial acetic acid treatment [41]. Literature suggests that the lactone variants formed and their distinct hydrolytic stability mainly depends on the rest of the glycoconjugate [39, 41]. From the current data it remains unclear whether a potential C4 lactone can be amidated via direct aminolysis with the same efficiency as the C2 lactone. Therefore, the extrapolation of the here presented results on sialyllactose to more complex glycoconjugates is not straightforward and requires the investigation of a wider variety of pure glycoconjugate standards by NMR instead.

## Conclusions

In this report, we investigated the lactone intermediate and its role in the amidation of α2,3-linked sialic acids in one-pot linkage-specific sialic acid derivatization workflows. We demonstrated that the amidation of α2,3-linked sialic acids occurs predominantly by direct aminolysis, and to a minor and likely negligible extent via the reaction of the free carboxyl group in the presence of a carboxylic acid activator and catalyst. We believe this report resolves the former ambiguity around the structure and role of the lactone intermediate in one-pot, in-solution, linkage-specific sialic acid derivatization reactions. As a consequence, this type of protocols has the potential to be significantly shortened and the addition of carboxylic acid activators in the second step of these reactions can be omitted.

## Supplementary Information

ESM 1 (PDF 2.88 mb)

ESM 2 (XLSX 400 kb)

## Data Availability

The datasets generated during and/or analyzed during the current study are available from the corresponding author on reasonable request.
