# Supplementary material for: The structure and role of lactone intermediates in linkage-specific sialic acid derivatization reactions
Source: Glycoconj J. 2021 Jan 18;38(2):157–66. doi: 10.1007/s10719-020-09971-7 (PMC8052245; doi:10.1007/s10719-020-09971-7)
Supplement: Supplementary file 1 — (PDF 2.88 mb) [file 10719_2020_9971_MOESM1_ESM.pdf]

SUPPORTING INFORMATION

Glycoconjugate journal

The structure and role of lactone intermediates in linkage-specific sialic acid derivatization reactions

Tamas Pongracz[1], Aswin Verhoeven[1], Manfred Wuhrer[1], Noortje de Haan[1,*,†]

[1]Center for Proteomics and Metabolomics, Leiden University Medical Center, 2333ZA Leiden, The Netherlands

[*]Corresponding author

t.pongracz@lumc.nl; a.verhoeven@lumc.nl; m.wuhrer@lumc.nl; ndehaan@sund.ku.dk

Correspondence: ndehaan@sund.ku.dk

[†]Copenhagen Center for Glycomics, University of Copenhagen, 2200 Copenhagen, Denmark

Table of contents:

Supplementary Fig. 1. MALDI-TOF-MS spectra **(a, b, d, e, g, h, j and k)** and bar graphs **(c, f, i and l)** showing the desired carboxyl group modification and the by-product introduced on the reducing end.

Supplementary Fig. 2. MALDI-TOF-MS spectra of sodium borohydride reduced α2,3- **(a-c)** and α2,6-sialyllactitol **(b-d)**.

Supplementary Fig. 3. MALDI-FTICR-MS spectrum of **(a)** the isolation of the methyl esterified α2,3-sialyllactitol artifact at *m/z* 672.238 introduced during the reduction protocol and **(b)** its MALDI-FT-ICR-MS/MS confirmation.

Supplementary Fig. 4. MALDI-TOF-MS spectra showing the modification induced on α2,3-sialyllactitol performed under native **(a-c)**, direct **(d, e)** and control **(f-h)** EE conditions.

Supplementary Fig. 5. MALDI-TOF-MS spectra of the modification induced on α2,3-sialyllactitol performed under native **(a-c)**, direct **(d, e)** and control **(f-h)** DMA conditions.

Supplementary Fig. 6. MALDI-TOF-MS spectra of the modification induced on α2,6-sialyllactitol performed under native **(a-c)**, direct **(d, e)** and control **(f-h)** EE conditions.

Supplementary Fig. 7. MALDI-TOF-MS spectra of the modification induced on α2,6-sialyllactitol performed under native **(a-c)**, pre-basified **(d, e)** and control **(f-h)** DMA conditions.

Supplementary Fig. 8. Bar graphs illustrating the NH_3 amidation and $MeNH_2$ amidation step in EtOH **(a)** or DMSO **(b)** using an α2,6-sialyllactitol standard.

Supplementary Fig. 9. Representative MALDI-FTICR-MS spectra of complex type *N*-glycans deriving from rhEPO under the tested conditions **(a-e)**.

Supplementary Fig. 10. Representative MALDI-FTICR-MS spectra of complex type *N*-glycans deriving from IgG under the tested conditions **(a-e)**.

Supplementary Fig. 11. Reaction products observed for α2,6-linked *N*-glycans from IgG under the tested conditions.

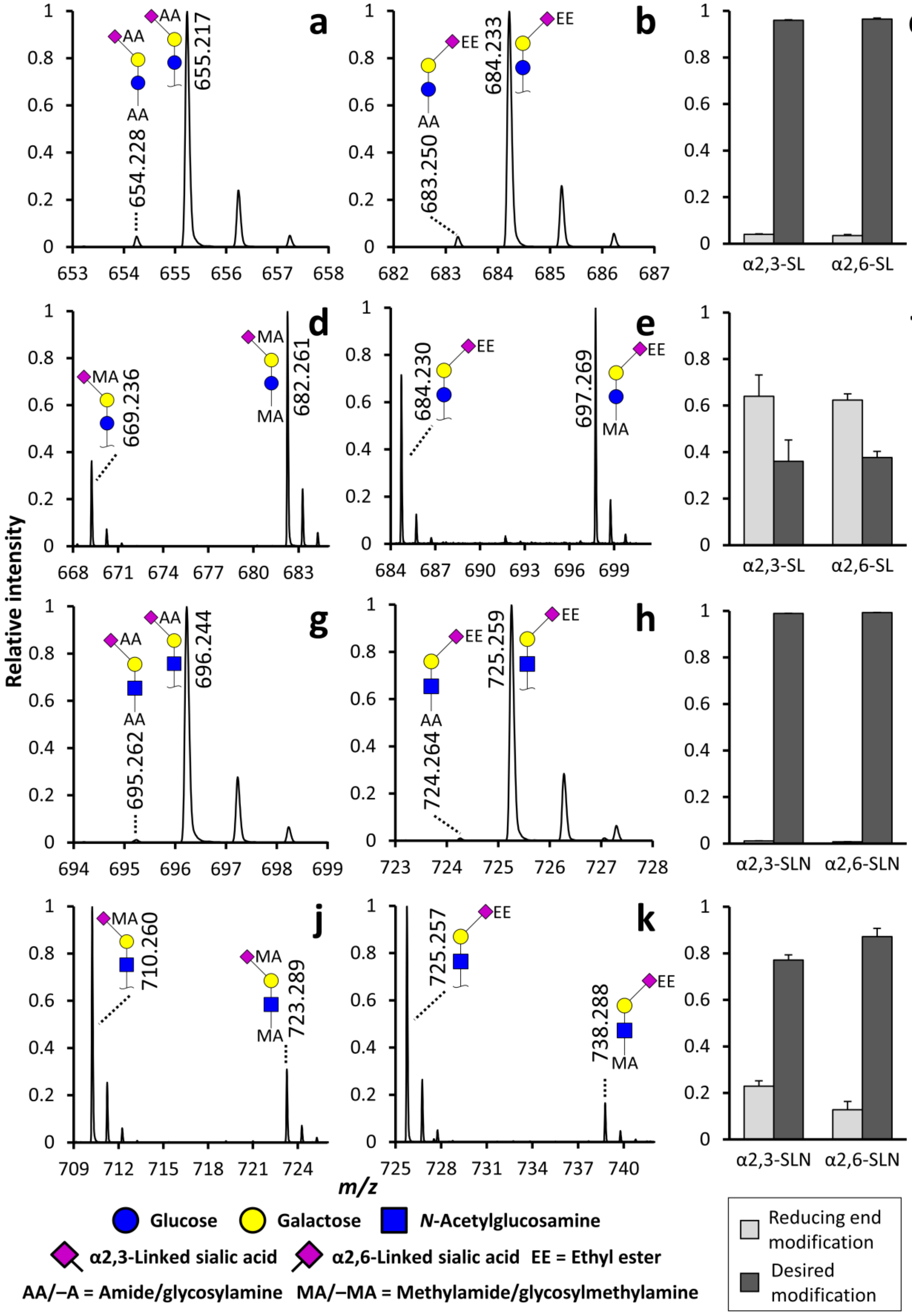

Supplementary Fig. 1. MALDI-TOF-MS spectra **(a, b, d, e, g, h, j and k)** and bar graphs **(c, f, i and l)** showing the desired carboxyl group modification and the by-product introduced on the reducing end. The glycosylamine introduced on the NH_3 amidated α2,3- **(a)** and ethyl esterified α2,6-sialyllactose **(b)**

or NH_3 amidated α2,3- **(g)** and ethyl esterified α2,6-sialylLacNAc **(h)** was observed at *m/z* -0.984 from the peak with the desired modification. The glycosylmethylamine introduced on the $MeNH_2$ amidated α2,3- **(d)** and ethyl esterified α2,6-sialyllactose **(e)**, or $MeNH_2$ α2,3- **(j)** and ethyl esterified α2,6-sialylLacNAc **(k)** was observed at *m/z* +13.032 from the peak with the expected modification. The α2,3-linkages are depicted with a left angle, with NH_3 amidation indicated by AA and $MeNH_2$ amidation by MA next to the sialic acid residue. The α2,6-linkages are depicted with a right angle, with ethyl esterification indicated by EE next to the sialic acid residue. Reducing end modifications are indicated as –AA (glycosylamine) and –MA (glycosylmethylamine). All species were detected as $[M+Na]^+$.

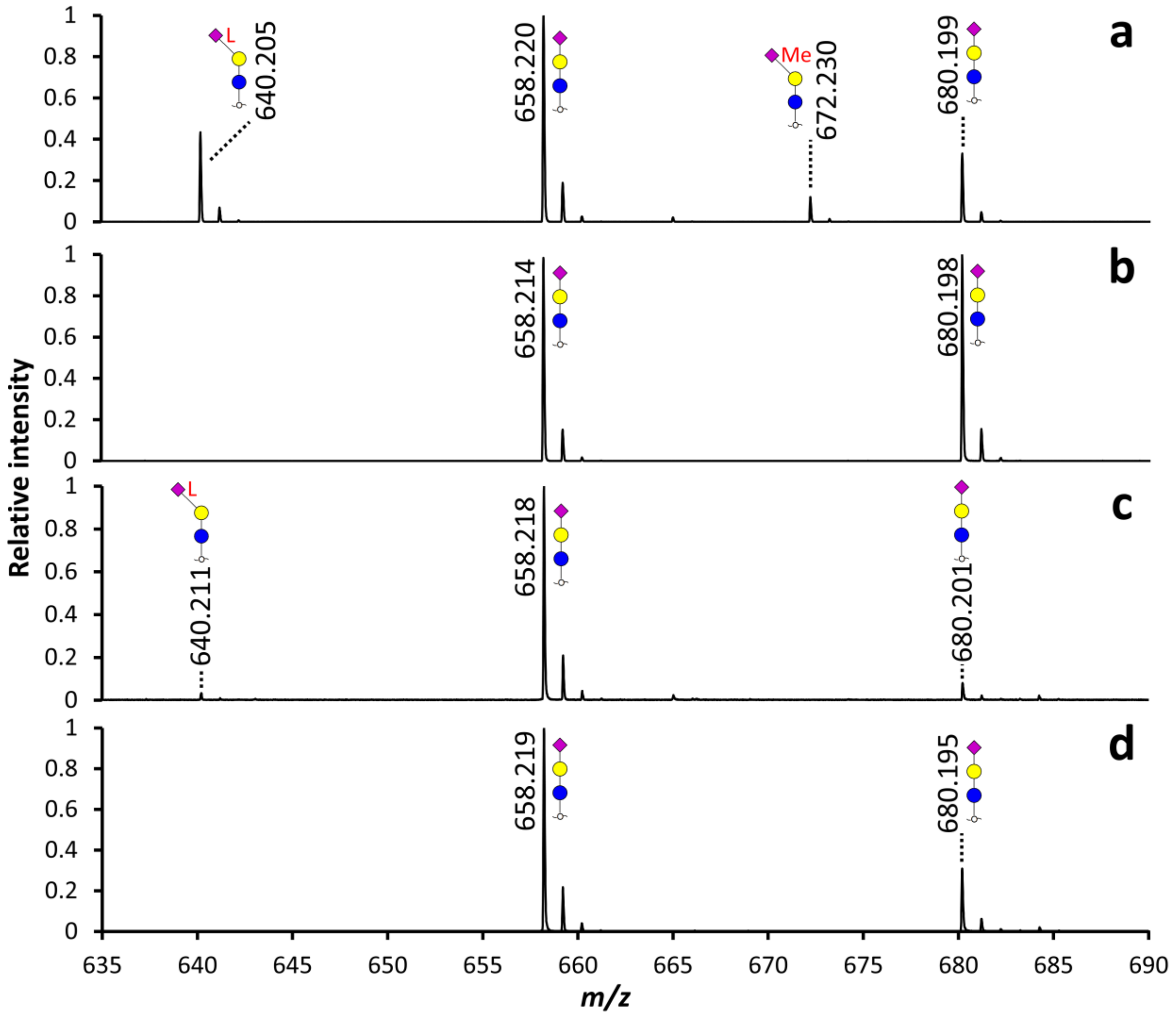

Supplementary Fig. 2. MALDI-TOF-MS spectra of sodium borohydride reduced α2,3- **(a-c)** and α2,6-sialyllactitol **(b-d).** Methanol **(a-b)** or isopropanol **(c-d)** was used for bead conditioning before cation-exchange clean-up and as co-evaporating agent during three consecutive borate removal steps in the SpeedVac concentrator. In case of derivatized sialic acids, an α2,3-linkage is depicted with a left angle, with undesired modifications indicated by L (lactonization), and Me (methyl esterification) in red next to the sialic acid residue. Non-derivatized sialic acids are depicted without an angle, and were detected as both $[M+Na]^+$ and $[M-H+2Na]^+$.

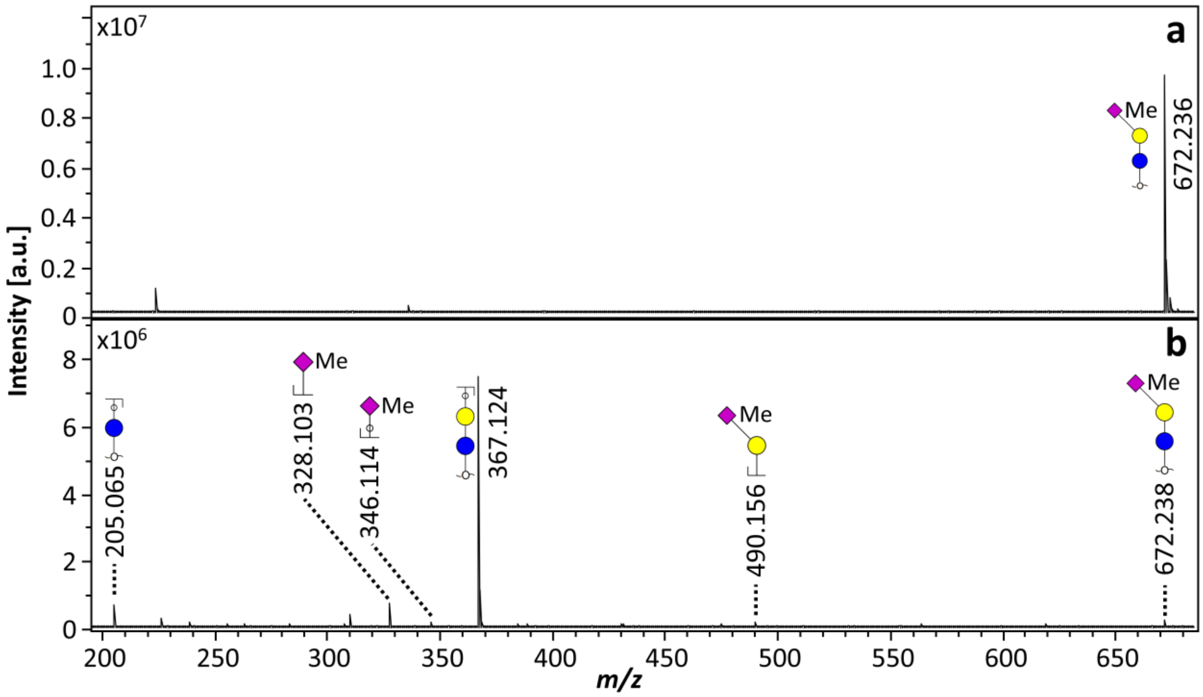

Supplementary Fig. 3. MALDI-FTICR-MS spectrum of **(a)** the isolation of the methyl esterified α2,3-sialyllactitol artifact at *m/z* 672.238 introduced during the reduction protocol and **(b)** its MALDI-FT-ICR-MS/MS confirmation.

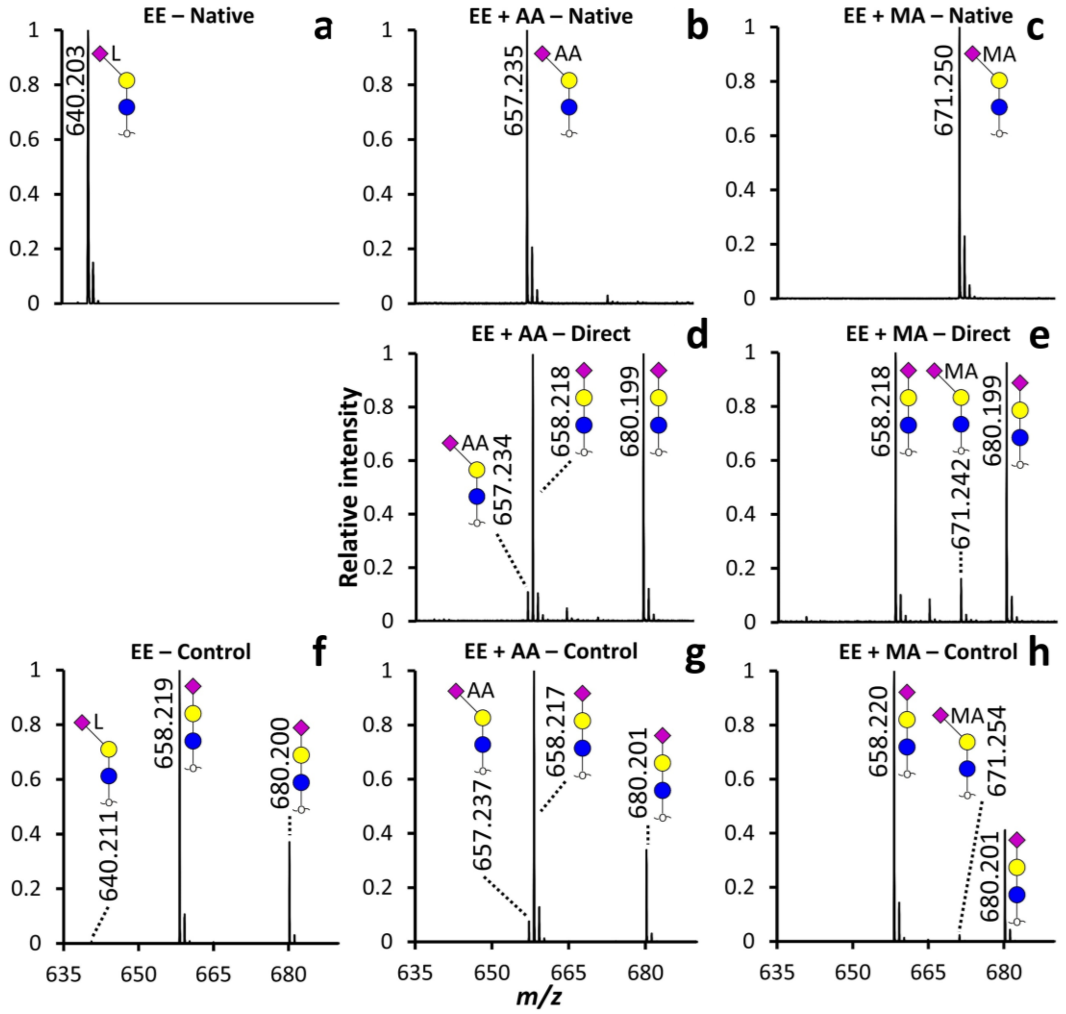

Supplementary Fig. 4. MALDI-TOF-MS spectra showing the modification induced on α2,3-sialyllactitol performed under native **(a-c),** direct **(d, e)** and control **(f-h)** EE conditions. In case of derivatized sialic acids, an α2,3-linkage is depicted with a left angle, with lactonization indicated by L, NH_3 amidation by AA, and $MeNH_2$ amidation by MA next to the sialic acid residue. Non-derivatized sialic acids are depicted without an angle, and were detected as both $[M+Na]^+$ and $[M-H+2Na]^+$.

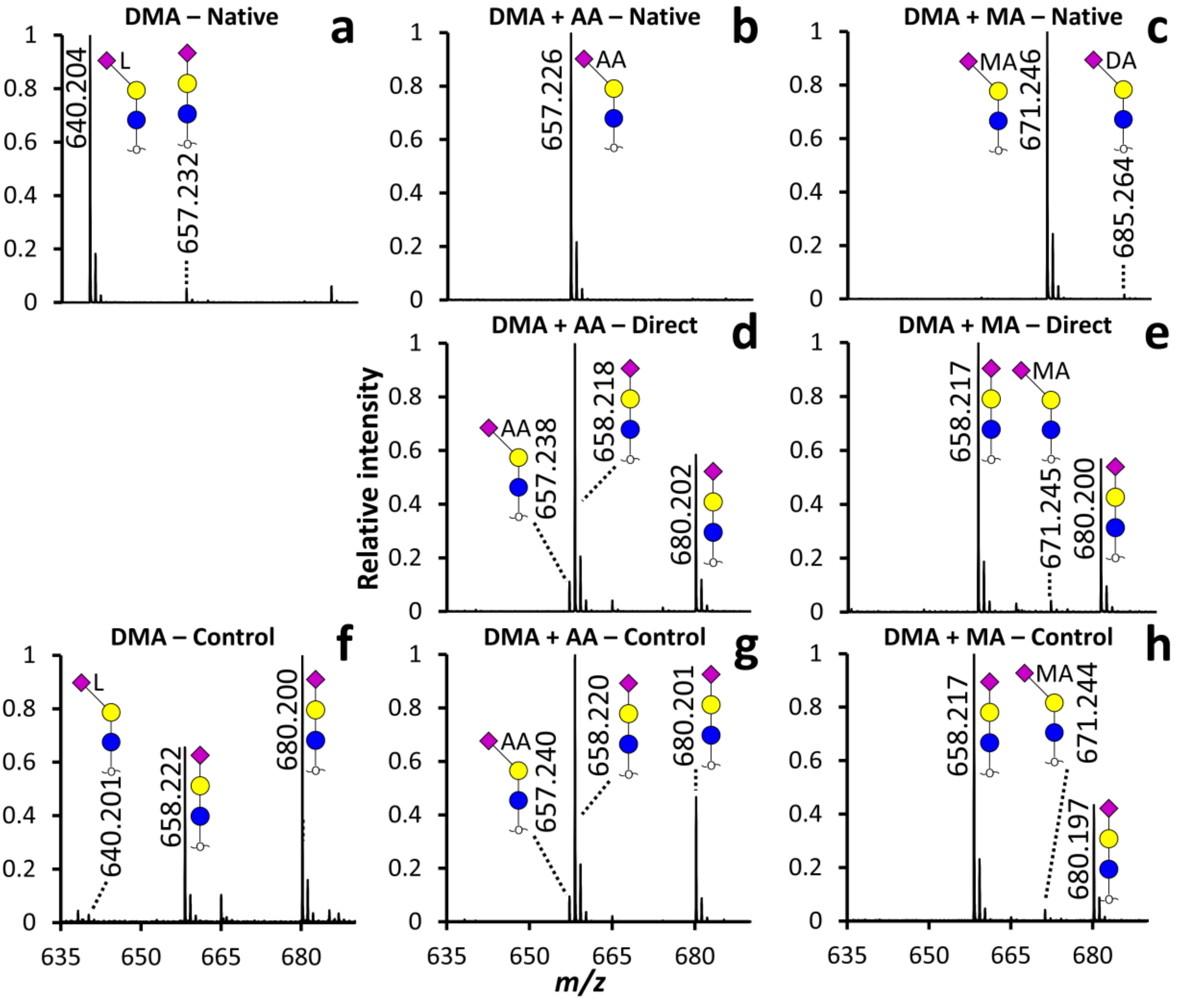

Supplementary Fig. 5. MALDI-TOF-MS spectra of the modification induced on α2,3-sialyllactitol performed under native **(a-c),** direct **(d, e)** and control **(f-h)** DMA conditions. In case of derivatized sialic acids, an α2,3-linkage is depicted with a left angle, with lactonization indicated by L, NH_3 amidation by AA and $MeNH_2$ amidation by MA next to the sialic acid residue. Non-derivatized sialic acids are depicted without an angle, and were detected as both $[M+Na]^+$ and $[M-H+2Na]^+$.

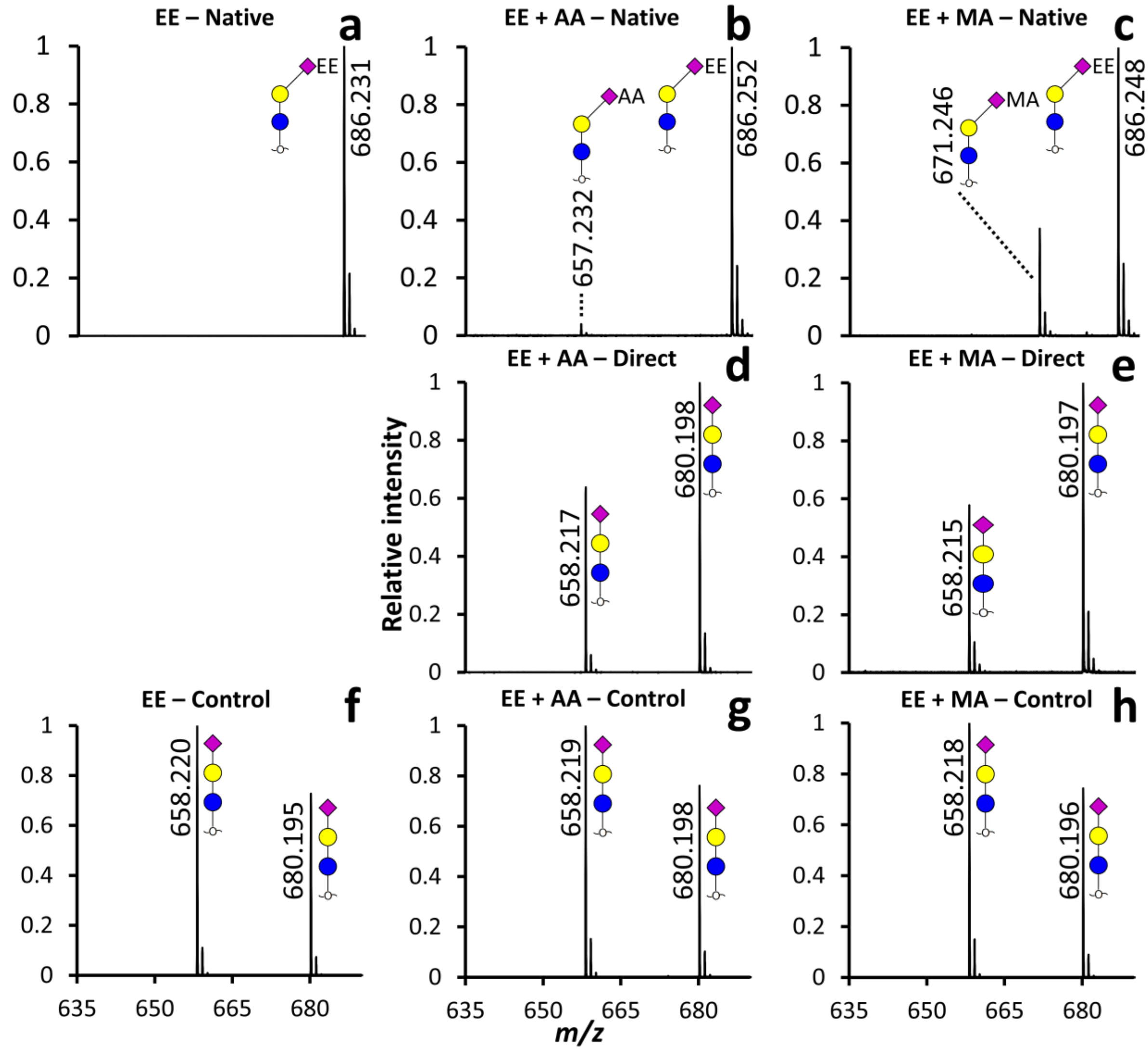

Supplementary Fig. 6. MALDI-TOF-MS spectra of the modification induced on α2,6-sialyllactitol performed under native **(a-c),** direct **(d, e)** and control **(f-h)** EE conditions. In case of derivatized sialic acids, an α2,6-linkage is depicted with a right angle, with NH_3 amidation indicated by AA, $MeNH_2$ amidation by MA and ethyl esterification by EE next to the sialic acid residue. Non-derivatized sialic acids are depicted without an angle. Non-derivatized sialic acids were detected as both $[M+Na]^+$ and $[M-H+2Na]^+$.

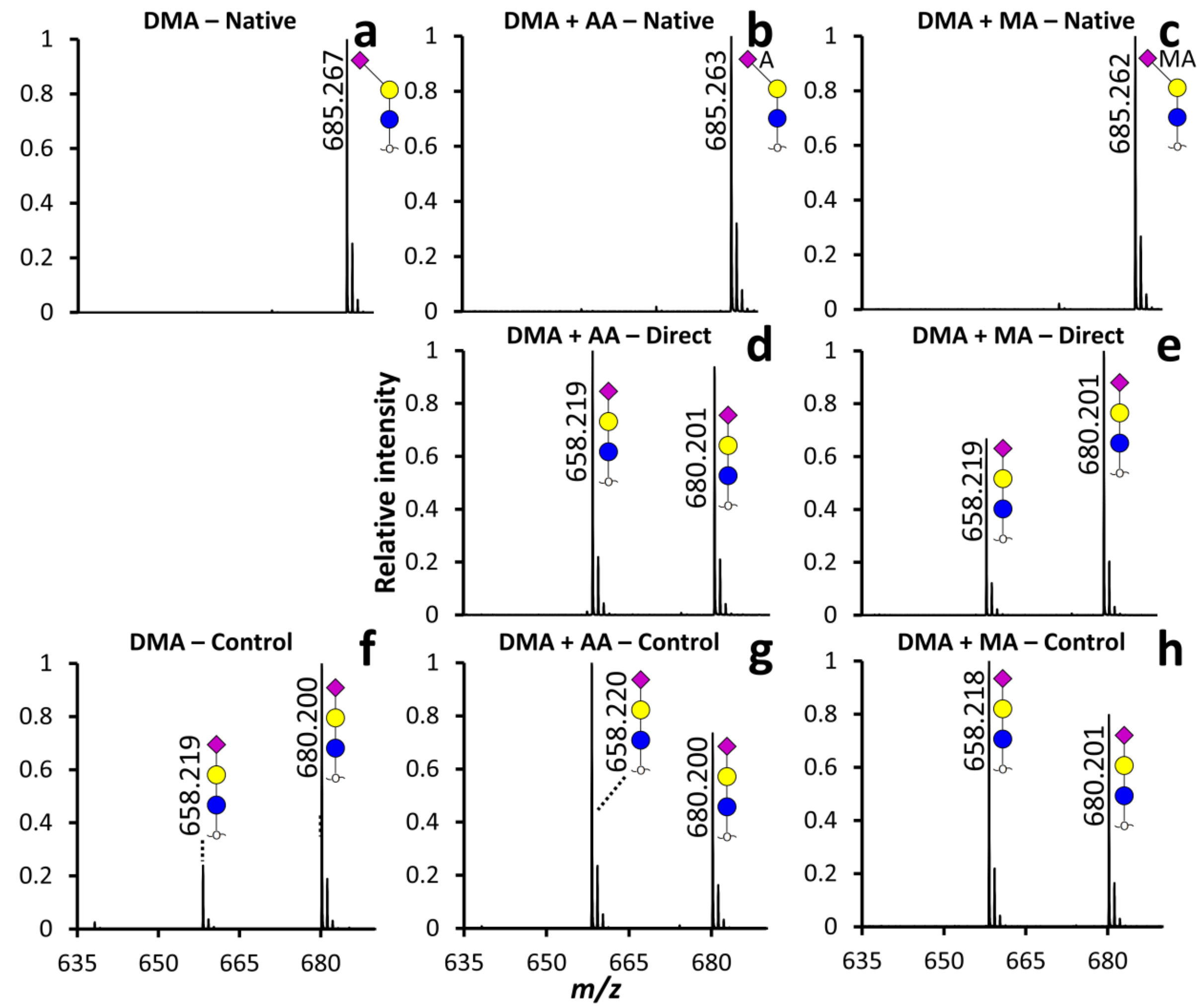

Supplementary Fig. 7. MALDI-TOF-MS spectra of the modification induced on α2,6-sialyllactitol performed under native **(a-c),** pre-basified **(d, e)** and control **(f-h)** DMA conditions. In case of derivatized sialic acids, an α2,6-linkage is depicted with a right angle, with NH_3 amidation indicated by AA, $MeNH_2$ amidation by MA and ethyl esterification by EE next to the sialic acid residue. Non-derivatized sialic acids are depicted without an angle, and were detected as both $[M+Na]^+$ and $[M-H+2Na]^+$.

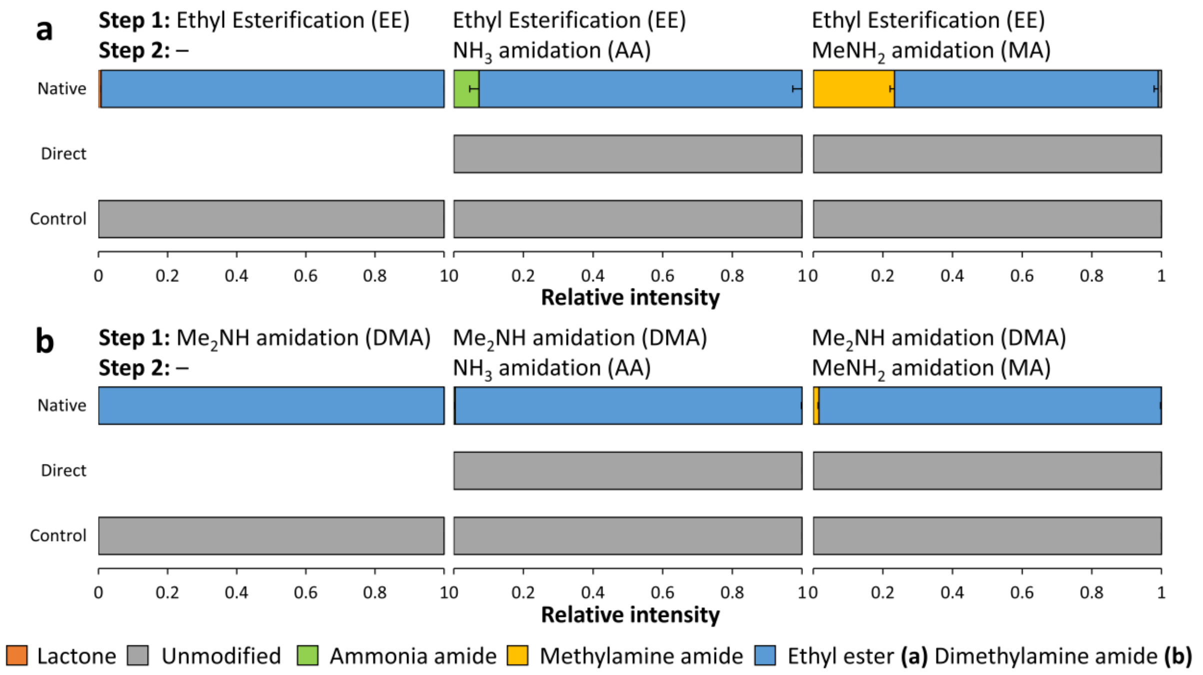

Supplementary Fig. 8. Bar graphs illustrating the NH_3 amidation and $MeNH_2$ amidation step in EtOH **(a)** or DMSO **(b)** using an α2,6-sialyllactitol standard. Native reaction conditions refer to the application of either EE or DMA reagent in the first step of the reaction and the addition of NH_3 or $MeNH_2$ in the second step. The direct conditions mimic the NH_3 amidation or $MeNH_2$ amidation step by the immediate addition of NH_4OH or $MeNH_2$ in the first step. The control conditions refer to the use of control-reagents without EDC and HOBt following the two-step reaction. The average and SDs for triplicate measurements are shown as stacked bars and error bars, respectively. Total area normalization was performed on the indicated species following data curation **(Supplementary Table 6)**. The pH of the conditions was evaluated in triplicate using narrow range pH indicator strips **(Supplementary Table 1)**.

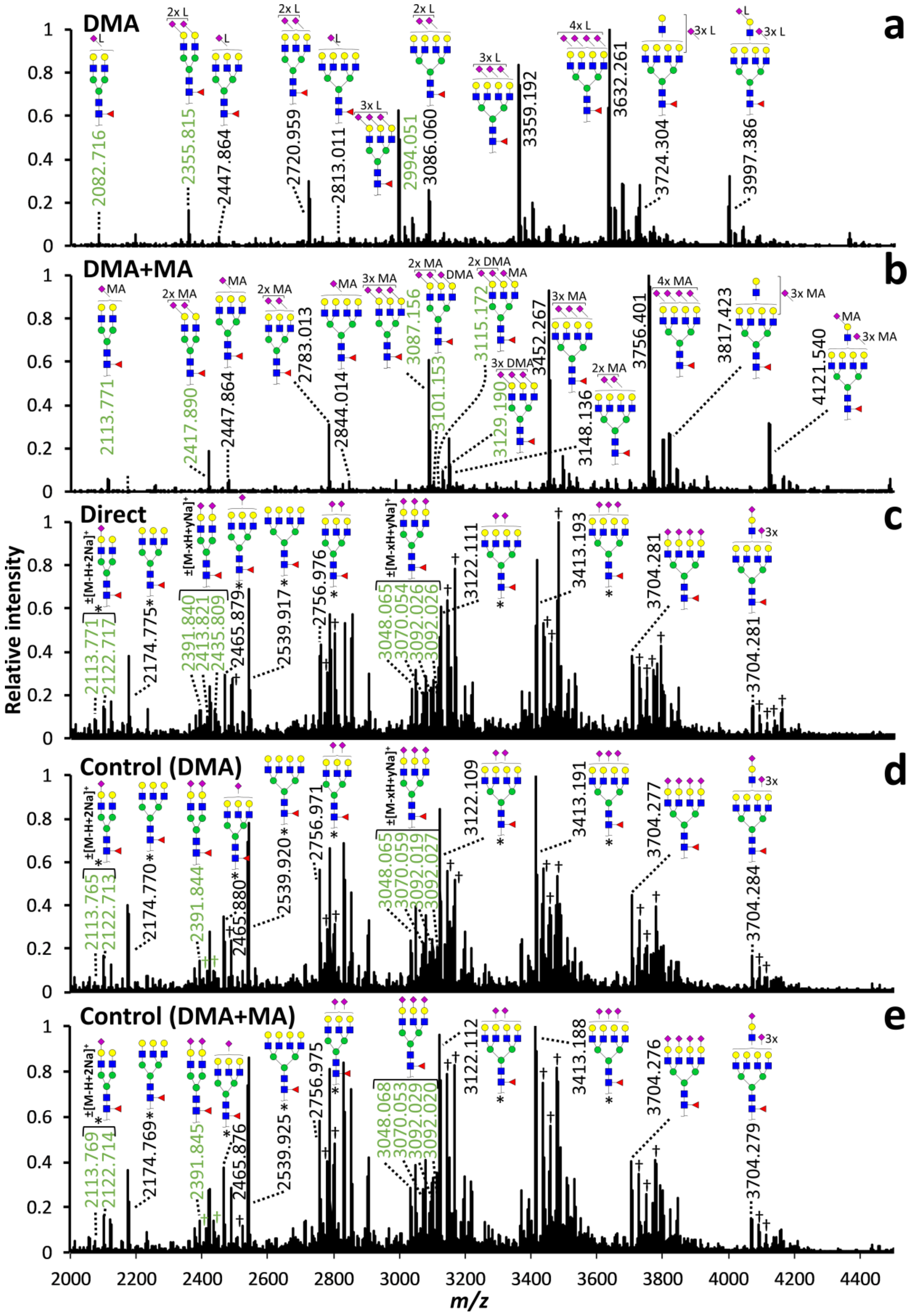

DMA
a
L
2x L
3x L
4x L
3x L
2082.716
2355.815
2447.864
2720.959
2813.011
2994.051
3086.060
3359.192
3632.261
3724.304
3997.386
DMA+MA
b
MA
2x MA
3x MA
4x MA
DMA
2x DMA
3x DMA
2113.771
2417.890
2447.864
2783.013
2844.014
3087.156
3101.153
3115.172
3129.190
3148.136
3452.267
3756.401
3817.423
4121.540
Direct
c
±[M-H+2Na]+
±[M-xH+yNa]+
2113.771
2122.717
2174.775
2391.840
2413.821
2435.809
2465.879
2539.917
2756.976
3048.065
3070.054
3092.026
3092.026
3122.111
3413.193
3704.281
3704.281
3x
Control (DMA)
d
2113.765
2122.713
2174.770
2391.844
2465.880
2539.920
2756.971
3048.065
3070.059
3092.029
3092.027
3122.109
3413.191
3704.277
3704.284
Control (DMA+MA)
e
2113.769
2122.714
2174.769
2391.845
2465.876
2539.925
2756.975
3048.068
3070.053
3092.019
3092.020
3122.112
3413.188
3704.276
3704.279
Relative intensity
m/z

Supplementary Fig. 9. Representative MALDI-FTICR-MS spectra of complex type *N*-glycans deriving from rhEPO under the tested conditions **(a-e).** In case of derivatized sialic acids, an α2,3-linkage is depicted with a left angle, with lactonization indicated by L, $MeNH_2$ amidation by MA, and Me_2NH amidation by DA next to the sialic acid residue. Non-derivatized sialic acids are depicted without an angle. The x indicator in the (mass definer) is a multiplier as 1, 2 or 3, while the y indicator in the (mass definer) is a multiplier as 2, 3 or 4. The *m/z* values highlighted in green indicate structures that have been selected for visualization in **(Fig. 5)**. Glycans labelled with an asterisk (*) denote potential in-source decay fragments. Peaks labelled with a cross (†) denote sialylated glycan signals that derive from neighboring peaks that underwent multiple carboxylic proton-to-sodium exchanges and are not depicted due to spatial constraints.

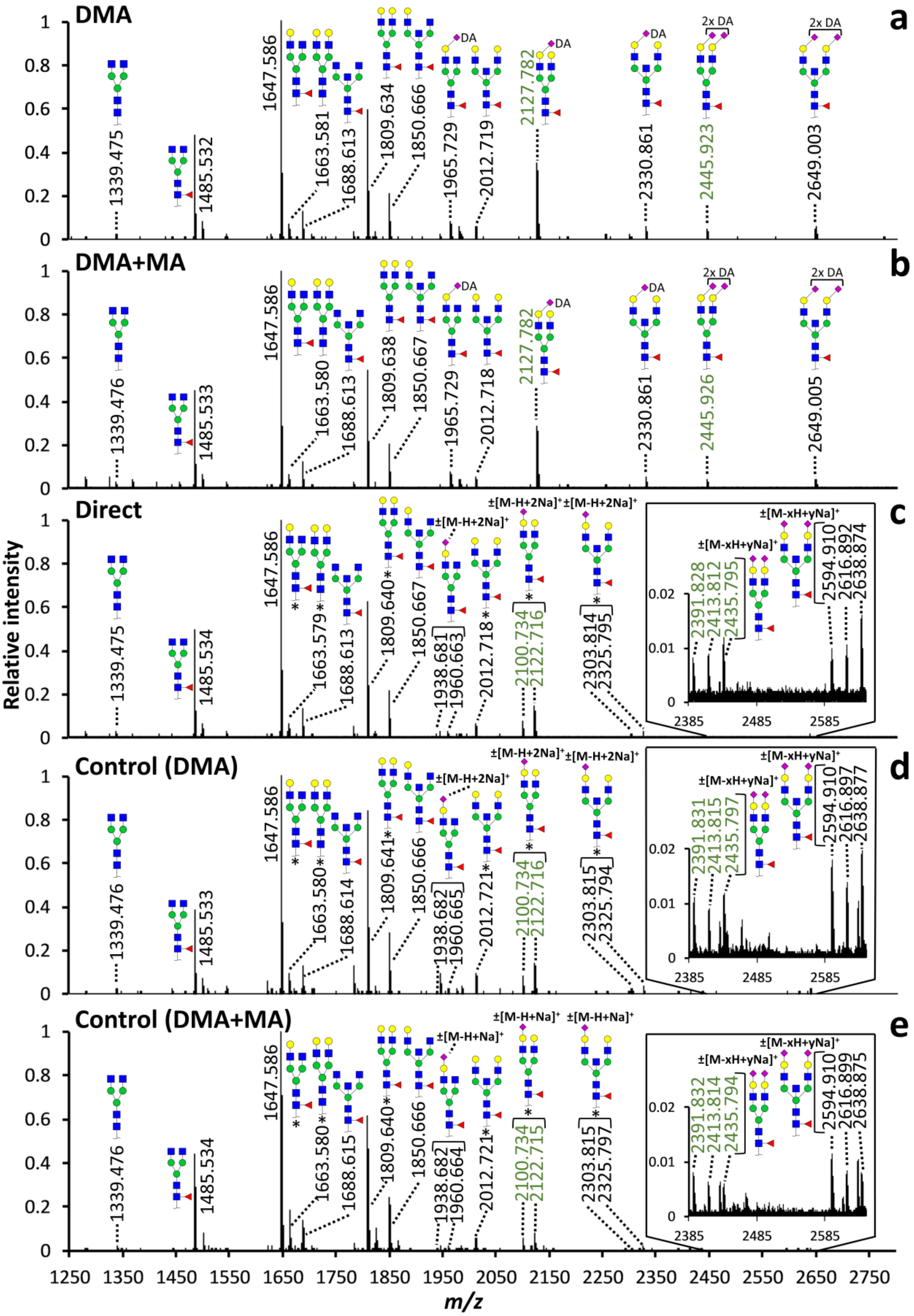

Supplementary Fig. 10. Representative MALDI-FTICR-MS spectra of complex type *N*-glycans deriving from IgG under the tested conditions **(a-e).** In case of derivatized sialic acids, an α2,6-linkage is depicted with a right angle, with Me_2NH amidation indicated by DA next to the sialic acid residue.

The x indicator in the (mass definer) is a multiplier as 1 or 2, while the y indicator in the (mass definer) is a multiplier as 2 or 3. The *m/z* values highlighted in green indicate structures that have been selected for visualization in **(Supplementary Fig. 11)**. Insets **(c-e)** show the *m/z* range of 2385-2650. No indication for mis-conversion or under-modification has been observed **(a, b)**. Glycans labelled with an asterisk (*) denote potential in-source decay fragments.

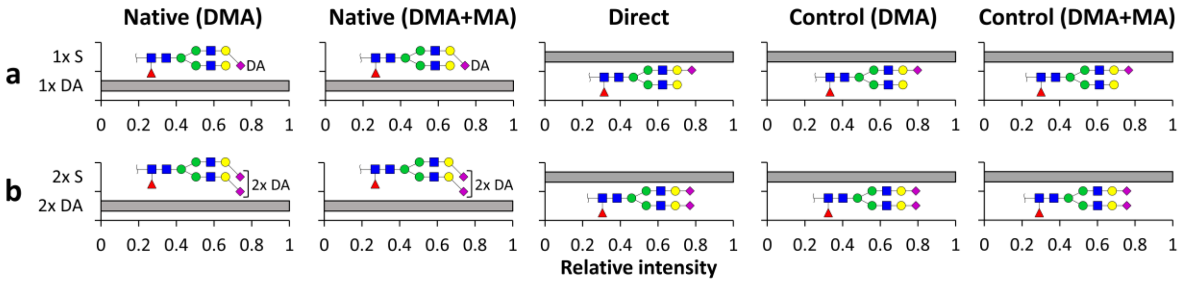

Supplementary Fig. 11. Reaction products observed for α2,6-linked *N*-glycans from IgG under the tested conditions. **(a)** H5N4F1S1 and **(b)** H5N4F1S2 are the gross glycan compositions evaluated. Abbreviations indicate H (hexose); N (*N*-acetylgalactosamine); F (fucose); S (*N*-acetylneuraminic acid). Symbols indicate the monosaccharide residues mannose (green circle), galactose (yellow circle) and *N*-acetylneuraminic acid (purple diamond). In case of derivatized sialic acids, an α2,6-linkage is depicted with a right angle, with Me_2NH amidation indicated by DA next to the sialic acid residue. Non-derivatized α2,6-sialic acids are depicted without an angle. The average and SDs for triplicate measurements are shown as bars and error bars, respectively. Total area normalization was performed on the indicated species following data curation **(Supplementary Table 7)**.
